# Extracranial versus intracranial hydro-hemodynamics during aging: a PC-MRI pilot cross-sectional study

**DOI:** 10.1186/s12987-019-0163-4

**Published:** 2020-01-14

**Authors:** Armelle Lokossou, Serge Metanbou, Catherine Gondry-Jouet, Olivier Balédent

**Affiliations:** 1 CHIMERE EA 7516 Research Team for Head & Neck, University of Picardie Jules Verne, CHU Amiens Sud, Bâtiment TEP 1er Étage, Unité de Traitement de l’image Médicale, Avenue René Laënnec, 80054 Amiens, France; 2 Department of Radiology, University Hospital, Amiens, France; 3 Department of Image Processing, University Hospital, Amiens, France

**Keywords:** CSF flow, Arterial cerebral blood flow, Venous cerebral blood flow, PC-MRI, Aging, Pulsatility

## Abstract

**Background:**

Both aging and changes in blood flow velocity between the extracranial (intraspinal) and intracranial regions of cerebral vessels have an impact on brain hydro-hemodynamics. Arterial and venous cerebral blood flows interact with cerebrospinal fluid (CSF) in the both the cranial and spinal systems. Studies suggest that increased blood and CSF flow pulsatility plays an important role in certain neurological diseases. Here, we investigated the changes in blood-CSF flow pulsatility in the cranial and spinal systems with age as well as the impact of the intracranial compartment on flow patterns.

**Method:**

Phase-contrast magnetic resonance imaging (PC-MRI) was performed in 16 young and 19 elderly healthy volunteers to measure the flows of CSF and blood. CSF stroke volume (SV), blood SV, and arterial and venous pulsatility indexes (PIs) were assessed at intra- and extracranial levels in both samples. Correlations between ventricular and spinal CSF flow, and between blood and CSF flow during aging were also assessed.

**Results:**

There was a significant decrease in arterial cerebral blood flow and intracranial venous cerebral blood flow with aging. We also found a significant increase of intracranial blood SV, spinal CSF SV and arterial/venous pulsatility indexes with aging. In regard to intracranial compartment impact, arterial and venous PIs decreased significantly at intracranial level in elderly volunteers, while young adults exhibited decrease in venous PI only. Intracranial venous PI was paradoxically lower than extracranial venous PI, regardless of age. In both sample groups, spinal CSF SV and aqueductal CSF SV were positively correlated, and so were extracranial blood and spinal CSF SVs.

**Conclusion:**

The study demonstrates that aging changes blood flow but preserves blood and CSF interactions. We also showed that many parameters related to blood and CSF flows differ between young and elderly adults.

## Introduction

Since the development of Phase Contrast Magnetic Resonance Imaging (PC-MRI), it is possible to assess the interaction between blood and CSF flow in the cranio-spinal system. The brain is contained in the rigid skull and subject to biomechanical constraint. This is especially relevant for intracranial pressure (ICP) which needs to be in physiological range to ensure good brain tissue perfusion. The Monro-Kellie doctrine states that, at steady state, the intracranial volume (brain, CSF, venous and arterial blood) is constant. In contrary, at each cardiac cycle, the brain, is subject to pulsatile arterial inflow that leads to CSF oscillations and venous drainage which in turn cause ICP changes [1]. Hence, cranio-spinal pulsatility is defined as the change in intracranial volume and pressure that occurs periodically with the cardiac cycle. Invasive procedures using ICP monitoring and infusion tests are used to study CSF dynamics in various conditions [2]. Non-invasive approaches are increasingly used to study cranio-spinal pulsatility [3, 4]. For example, stroke volume, i.e. the mean volume of fluid that oscillates during the cardiac cycle, is used to assess blood and CSF pulsatility between different compartments [3]. However, the use of aqueductal CSF stroke volume as a diagnostic marker of ICP alteration is still debated. For the vascular system, pulsatility or resistance indexes of cerebral blood flow are used as indirect measures for the biomechanical properties of the vascular tree and blood pressure [5, 6].

Using PC-MRI, some authors showed increased cerebral arterial pulsatility with aging [7, 8]. Zarrinkoob et al. further showed that the ratio between the extracranial or spinal proximal and the intracranial distal artery pulsatility indices, called the flow dampening factor, is lower for older people than for the younger ones [8]. These authors attributed this result to the increasing flow pulsatility with increasing age, as a result of stiffness of the vessels when elastin is replaced by collagen [8]. In recent studies, Schubert et al. showed that arterial blood flow pulsatility decreases from the distal to proximal part of the vessels and that this reduced pulsatility increased with the tortuosity of the vessels at the level of the atlas or of the carotid siphon [6, 9]. A study by Stoquart-Elsankari et al. in healthy young adults showed that jugular venous flow pulsatility is higher than the superior sagittal sinus flow pulsatility [10].

Some studies have also suggested a role for flow pulsatility in some neurological conditions. Indeed, pulse wave encephalopathy is a term associated with disorders such as Alzheimer’s disease, vascular dementia, and hydrocephalus. The pulse wave encephalopathy theory states that there is an underlying vascular pathophysiology behind these conditions which is related to the strength of arterial pulse waves induced in the cranio-spinal cavity [11]. Bateman showed an increase of 56% of arterial pulsatility and 70% of sagittal sinus pulsatility in hydrocephalus patients compared to patients with dementia [12]. El Sankari et al. showed an increase of vascular pulsatility indexes in amnesic patients compared to healthy subjects [13]. Furthermore, Lim et al. studied the pulsatility of anterior, middle and posterior cerebral arteries and cognitive functions in patients with middle to moderate Alzheimer’s disease. They found that the highest values of pulsatility were associated with poor scores after neuropsychology tests and that the rise of these pulsatilities 1 year after was associated with disease progression [14]. However, to date, unlike the diminution of white and grey matter during physiological aging which is well-documented [15, 16], the physiological aging effect on cerebral blood and CSF flow patterns and their relationship is poorly investigated.

As age influences arterial flow pulsations, we hypothesized that age would influence the venous flow pulsations and the blood-CSF interactions. The objective was to assess the effects of the intracranial compartment and age on cranio-spinal flows and their pulsatilities, as well as on the interplay between CSF and vascular flows.

## Materials and methods

### Study population

In this study, we used both elderly and younger populations to account for the age variation. The elderly population, defined as Healthy Elderly Volunteers (HEV), consisted of 19 elderly subjects (13 women and 6 men). Their mean age was 73 ± 6 years. They were all recruited to the Neurology department of our hospital and examined by a neurologist with 10 years’ of experience to exclude seizures, transient neurological deficits, or gait disorders. Exclusion criteria were: cognitive decline (all subjects underwent a neuropsychological screening with a Mini-Mental State Examination [MMSE] and score greater than 26/30), a relevant cerebral neurological disease (cerebrovascular accident, meningoencephalitis, tumor), or relevant cerebrovascular risk factors, except arterial hypertension controlled by medication. The healthy elderly volunteers underwent also magnetic resonance imaging (MRI) of the brain to exclude ventricular enlargement.

The younger population, here called Healthy Young Volunteers (HYV), consisted of 16 healthy young subjects (9 women and 7 men), who underwent cine phase-contrast MRI. Their mean age was 31 ± 7 years. The exclusion criteria were: any neurological, psychiatric or severe general disease, alcoholism, or abnormalities detected by a clinical MRI exam.

The study was approved by the local ethics review committee, and written informed consent was obtained from all subjects.

In this paper, HEV was used to designate subjects belonging to the elderly population, and HYV for subjects belonging to the young population.

### PC-MRI protocol

**Fig. 1:**
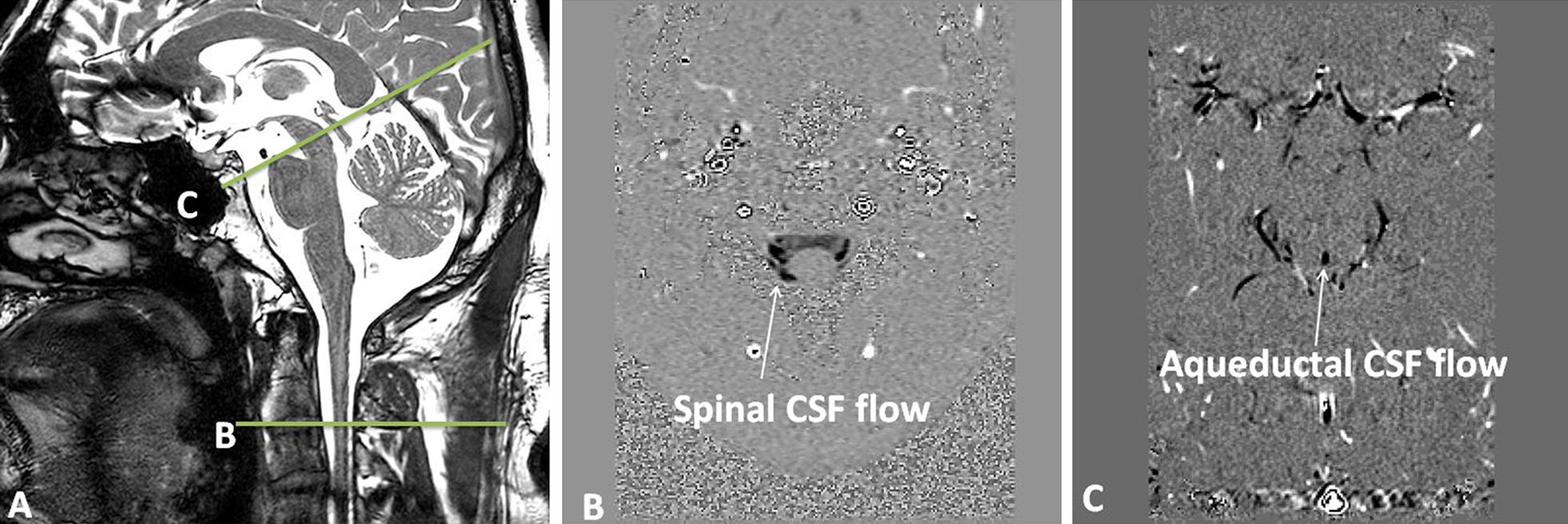
CSF flow acquisition imaging. Sagittal 3D Fiesta (Fast Imaging Employing Steady-state Acquisition) image (**a**) was used as a reference to set the selected acquisition plane perpendicular to the flow direction in CSF compartments at the C2–C3 level of the spine (**b**) and through the aqueduct (**c**)

**Fig. 2:**
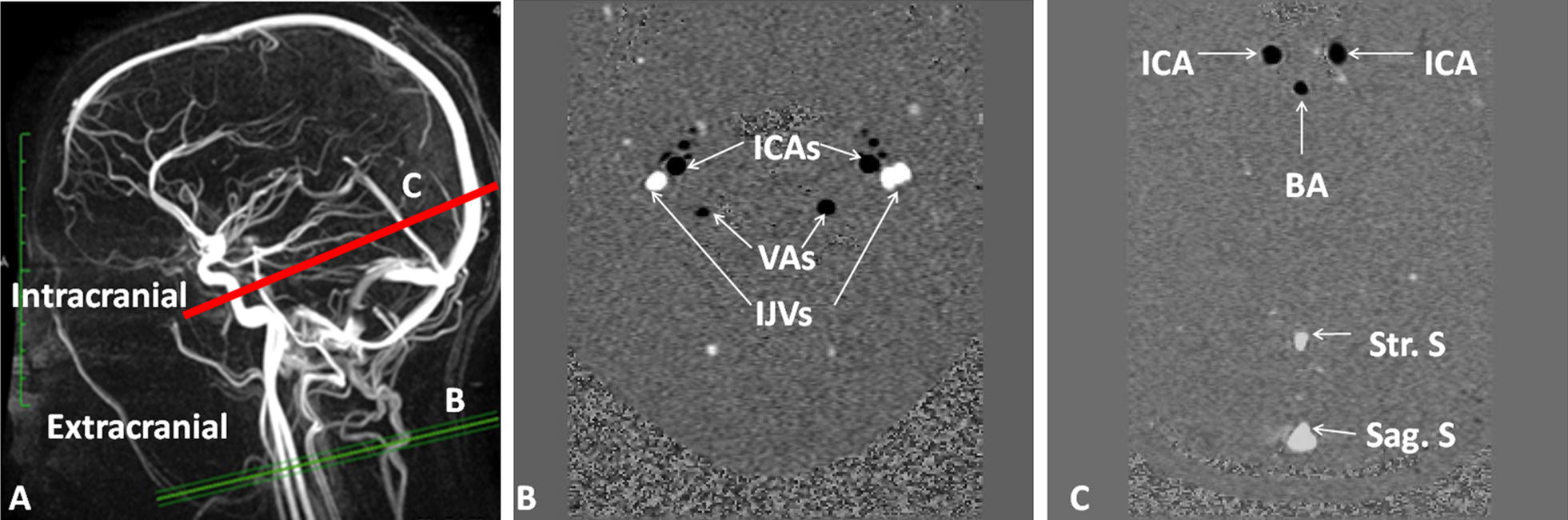
Extracranial and intracranial acquisition imaging. Sagittal 3D phase-contrast angiography (**a**) image was used as a reference to set the different acquisition planes perpendicular to the flow direction in the vessels. Extracranial acquisition (**b**) was used to quantify vascular flows in right and left internal carotid arteries (ICAs), vertebral arteries (VAs) and both internal jugular veins (IJV). Intracranial acquisition (**c**) was used to quantify vascular flows in right and left internal carotid arteries (ICAs), basilar artery (BA); sagittal sinus (Sag. S), and straight sinus (Str. S)

All exams were performed using 3T MRI (Signa HDx General Electric Medical System, Milwaukee, WI). The subjects were supine with their head in the 16-channel MRI head coil. Sagittal 3D Fiesta (Fast Imaging Employing Steady-state Acquisition) (Fig. 1a) and sagittal 3D phase-contrast angiography (Fig. 2a) were used as references to set the different planes for CSF and blood flow acquisitions, respectively. The duration of these sequences was approximately 3 min for sagittal 3D Fiesta and 5 min for 3D phase-contrast angiography.

The main settings for the sagittal 3D Fiesta sequence were: TE = 3 s; TR = 6 s; flip angle = 45°; matrix = 512 × 512; spatial resolution = 0.35 × 0.35 mm^2^; slice thickness = 1.2 mm; number of averages = 1.

For the 3D phase contrast sequence, the following settings were considered: TE = 3 s; TR = 5 s; flip angle = 12°; matrix = 288 × 288; spatial resolution = 1.22 × 1.22 mm^2^; slice thickness = 3 mm; number of averages = 1.

A regular heart rate (expressed in beats per minute: BPM) was required for all participants before starting PC-MRI acquisition sequences. This implied consistent curves of cardiac pulse in accordance with the subject’s heartbeat, which needed to be in the physiological range of 45–100 BPM [17]. Retrospective cardiac-gated PC-MRI sequences were performed with a plethysmograph set on the subject’s finger to synchronize the acquired images with the cardiac cycle.

During the acquisition, care was needed to set the acquisition planes perpendicular to the flow direction in the arterial and venous vessels and to the CSF flow direction in both CSF compartments. As shown in Fig. 1a, good morphological acquisition, highlighting CSF in the cranial and spinal compartments, was important in setting the slices. We followed the same approach for the vascular flow investigation in which 3D angiography (Fig. 2a) was used. This allowed for the visualization of the complex geometry of the vessels in different planes before starting the acquisition and ensured that the slice was orthogonal to the vessels. Indeed, as shown in Fig. 2a at the intracranial level, the internal carotid arteries are tortuous before the Willis polygon, so it was crucial to set the slice in the straight segment of the vessels.

For CSF flow acquisitions, spinal and aqueductal CSF flows were evaluated at the C2-C3 level of the spine (Fig. 1a) and at the aqueductal level (Fig. 1a), respectively. Extracranial acquisition (Fig. 2b) was used to quantify flows in the right and left internal carotid arteries (ICAs), both vertebral arteries (VAs) and both internal jugular veins (IJVs). Intracranial acquisition (Fig. 2a′) was used to quantify flows in the right and left ICAs, the basilar artery (BA), and sinuses (sagittal and straight).

The main settings for these PC-MRI sequences were defined according to previous work [18, 19]: TR and TE set at the minimum values, field of view: 140 mm × 140 mm, section thickness: 5 mm, flip angle: 25° for vascular flows and 20° for CSF flows, spatial resolution: 0.55 mm × 0.55 mm, and matrix: 256 × 128. Velocity encoding (V_enc_) was set at 80 cm/s for blood vessels and at 5 cm/s, and at 10 cm/sec for CSF flows at the spinal and aqueductal levels, respectively. Each cardiac cycle was sampled to thirty-two time points.

By convention, cranio-caudal flows are shown in white and caudo-cranial flows in black. Aliasing pixels are directed opposite to the flow in the region of interest. When more than half of the pixels in the region of interest presented velocity aliasing, the acquisition was repeated with double V_enc_ because the algorithm used in Flow software [1] can only correct 50% of aliasing in the region of interest. Otherwise, velocity aliasing was corrected (V_corrected_) during data processing as follows:

$${\text{V}}_{\text{corrected}} = \left( { 2 { } \times {\text{ V}}_{\text{enc}} - \, |{\text{V}}_{\text{error}} |} \right) \, \times \, \left( { - \left| {{\text{V}}_{\text{error}} } \right|/{\text{V}}_{\text{error}} } \right),$$

where Venc is the encoding velocity and V_error_ is the aliased velocity [1].

The duration of the cardiac period in the population was 0.89 ± 0.15 s and the duration of each sequence was approximately 105 ± 46 s.

Spatial resolution, orthogonality of the slice to the vessels and in the CSF compartments, and the minimum echo time were chosen to avoid the partial volume effect during data processing according to previous works [20, 21].

### PC-MRI data processing

PC-MRI data were analyzed with *Flow* software designed in our laboratory [1] to extract the flow curves for both the blood vessels and the CSF regions of interest (ROI). The *Flow* software used a semi-automated segmentation algorithm based on the properties of CSF and blood flow to calculate the areas of ROI and to determine the mean velocity of the fluid at each time point of the cardiac cycle. Then, the reconstruction of the mean flow curves was performed by multiplying the mean velocity curve by the area of the ROI [1].

The temporal algorithms in the *Flow* software make it possible to study very challenging regions such as the spinal CSF region, where subarachnoid spaces (SAS) are crossed by nerves dividing them into different channels. This makes the spinal CSF region more difficult to manually segment than the cerebral aqueductal region. Here, the data were analyzed by the same research team individual to minimize errors. Moreover, using this software, it has been demonstrated that volumetric flow measurements are reproducible with a normalized standard deviation of less than 5% [1].

**Fig. 3:**
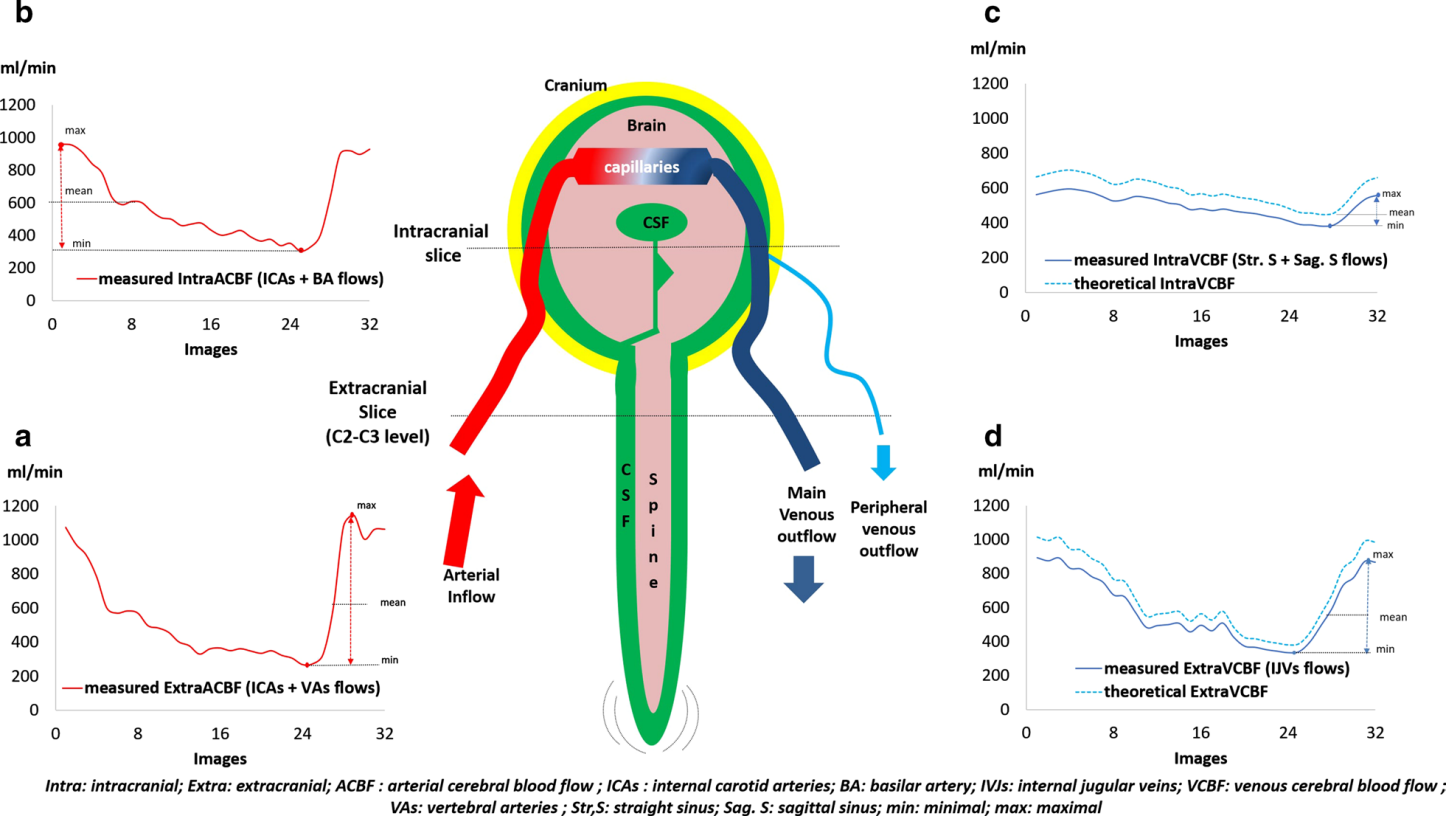
Examples of flow over one cardiac cycle in all the vessels which composed the total arterial and venous cerebral blood flows of one young volunteer. **a** measured extracranial arterial flow (internal carotid + vertebral), **b** measured intracranial arterial flow (internal carotid and basilar), **c** measured intracranial venous flow (straight and sagittal sinuses), **d** measured extracranial venous flow (internal jugular). Pulsatility was calculated from PC-MRI data obtained along the cardiac cycle, both at intracranial and extracranial levels. Theoretical venous cerebral blood flows represent the measured venous flows and were corrected by an *alpha* factor to account for the peripheral cerebral veins, which were not measured. For these 4 flows, a pulsatility index, PI = (max–min)/mean, was calculated

The total extracranial arterial cerebral blood flow (ExtraACBF) was calculated by adding the two internal carotid and the two vertebral artery flows (Fig. 3a). Similarly, the total intracranial arterial cerebral blood flow (IntraACBF) was calculated by adding both the internal carotid artery and basilar artery flows (Fig. 3b).

The measured extracranial venous cerebral blood flow (ExtraVCBF) was calculated from the flow in the internal jugular veins (Fig. 3d) and the measured intracranial venous cerebral blood flow (IntraVCBF) by adding the straight and sagittal sinus flows (Fig. 3c).

As we did not take into account the peripheral venous drainage, and considering that arterial cerebral blood flow must be equal to theoretical venous cerebral blood flow (main venous outflow + peripheral venous outflow), ExtraVCBF and IntraVCBF were corrected by multiplying ExtraVCBF and IntraVCBF by *α*Extra and αIntra, the venous correction factors to obtain a new theoretical venous cerebral blood flow (Fig. 3c, d). αExtra and αIntra were obtained, respectively, by dividing ExtraACBF and IntraACBF by ExtraVCBF and IntraVCBF, respectively [1].

The arteriovenous flow was calculated by subtracting the theoretical VCBF from ACBF at each time point in the cardiac cycle. This arteriovenous flow was integrated to calculate the total cerebral blood volume change during the cardiac cycle, the blood stroke volume (blood SV), both for extracranial (Extra blood SV), and intracranial levels (Intra blood SV). Aqueductal and spinal CSF flows were also reconstructed during the cardiac cycle.

Spinal CSF stroke volume (spinal CSF SV) and aqueductal CSF stroke volume (aqueductal CSF SV) were calculated as previously reported by taking into account the mean area under the curves of spinal CSF flow and aqueductal CSF flow [1].

A pulsatility index (PI) was defined from the maximal, minimal, and mean flow values (Fig. 3) for the intra- and extracranial arterial and venous cerebral blood flows:

$$PI = \frac{{{\text{Maximal flow}} - {\text{Minimal flow}}}}{\text{Mean flow}}$$

This PI was established initially in Doppler investigations [22] and has been used in several studies using PC-MRI [6, 8].

### Statistical analyses

The Shapiro test was used to evaluate the distribution of the different variables and test for their normality. In case of normal distribution, the parametric tests were used (Student t and Pearson correlation tests); otherwise, non-parametric tests (Mann–Whitney or Wilcoxon test) were performed. The results were considered significant at *p *< 0.05.

We compared extracranial and intracranial flow parameters for each group using paired tests. We also tested the differences between healthy young volunteers and healthy elderly volunteers using independent tests.

We excluded one young volunteer because we were missing the corresponding spinal CSF stroke volume value. We also excluded 2 aberrant values from the HYV subjects and 2 aberrant values from the HEV subject sample after analyzing the boxplots of the variables. These values were defined as aberrant when there were much higher or lower than the confidence interval (CI): ${\text{CI}} = \left[ {mean - 1.5 \times interquartile ; mean + 1.5 \times interquartile} \right]$ [23].

By screening the subjects individually, these values correspond to subjects who moved during MRI acquisition, and we believe that these aberrant values could come from large vessel motion during the cardiac cycle.

Finally, we used the Pearson correlation test to assess the relation between the stroke volumes. Correlations between 0.30 and 0.49 were considered weak, between 0.50 and 0.69 moderate, between 0.70 and 0.89 strong, and higher than 0.90 were very strong.

## Results

### Comparison between extra- and intracranial flow parameters within each group

The results of the comparison of the extra- and intracranial hydro-hemodynamic parameters in HYV versus HEV are summarized in Table 1. Particularly, we found that extra- and intracranial arterial cerebral blood flows (ACBF) did not differ significantly in HYV (716 ± 129 versus 670 ± 158 ml/min; p = 0.4; Table 1) or in HEV (588 ± 119 versus 593 ± 107 ml/min; p = 0.7; Table 1). Similarly, extra- and intracranial blood stroke volumes were not significantly different in HYV (0.91 ± 0.39 versus 0.76 ± 0.30 ml/cc) and in HEV (0.98 ± 0.32 versus 1.08 ± 0.21 ml/cc). Interestingly, measured extra- and intracranial venous cerebral blood flows were similar in the HYV group (449 ± 173 versus 478 ± 94 ml/min), but differed within the HEV group, with a significantly higher extracranial venous cerebral blood flow (533 ± 161 versus 379 ± 88 ml/min; p = 0.0002; see Table 1). The same patterns were also observed for the extra- and intracranial α venous correction values, which were similar in HYV (1.67 ± 0.75 versus 1.52 ± 0.34; p = 0.68; Table 1), but significantly different for the HEV group, with the intracranial value being significantly higher than that of the extracranial α venous correction (1.59 ± 0.41 versus 1.24 ± 0.52; p = 0.038). Extra- and intracranial arterial PI values did not differ significantly in HYV (0.85 ± 0.17 versus 0.77 ± 0.22; Table 1). A contrasting pattern was however observed within the HEV group, with the intracranial arterial PI being significantly lower than the extracranial arterial PI (1.16 ± 0.17 versus 1.31 ± 0.32; p = 0.003; Table 1). Intracranial venous PI was significantly lower compared to extracranial PI in both groups (HYV: 0.25 ± 0.16 versus 0.47 ± 0.25 and HEV: 0.52 ± 0.16 versus 0.86 ± 0.40; Table 1).

**Table 1:** Comparison of intracranial and extracranial flow parameters in younger and older healthy adults

Parameters	Healthy young volunteers mean ± SD (max–min)	Healthy elderly volunteers mean ± SD (max–min)	p-values
n	16	19	
Age	31 ± 7 (26–44)	73 ± 6 (63–82)	***
ExtraACBF (ml/min)	716 ± 129 (531–933)	588 ± 119 (396–771)	0.007**
IntraACBF (ml/min)	670 ± 158 (329–951)	593 ± 107 (343–753)	0.12^ns^
*p*-*values*	*0.4*^*ns*^	*0.7*^*ns*^	
Mean Total Arterial Cerebral Blood Flow (ml/min)	693 ± 117 (433–899)	591 ± 102 (370–740)	0.006**
ExtraVCBF (ml/min)	449 ± 173 (250–778)	533 ± 161 (171–707)	0.09^ns^
IntraVCBF (ml/min)	478 ± 94 (365–682)	379 ± 88 (171–501)	0.01*
*p*-*values*	*0.66*^*ns*^	0.0002***	
αExtra	1.67 ± 0.75 (0.95–3.71)	1.24 ± 0.52 (0.65–2.44)	0.01*
αIntra	1.52 ± 0.34 (0.99–2.38)	1.59 ± 0.41 (0.99–2.64)	0.81^ns^
*p-values*	*0.68*^*ns*^	0.038*	
Extra blood SV (ml/cc)	0.91 ± 0.39 (0.49–2.08)	0.98 ± 0.32 (0.48–1.61)	0.24^ns^
Intra blood SV (ml/cc)	0.76 ± 0.30 (0.29–1.28)	1.08 ± 0.21 (0.53–1.57)	0.001**
*p-values*	*0.21*^*ns*^	*0.31*^*ns*^	
Spinal CSF SV (ml/cc)	0.34 ± 0.21 (0.08–0.86)	0.51 ± 0.20 (0.16–0.89)	0.02*
Aqueductal CSF SV (ml/cc)	0.04 ± 0.02 (0.01–0.08)	0.05 ± 0.03 (0.01–0.10)	0.26^ns^
Extra arterial PI	0.85 ± 0.17 (0.45–1.11)	1.31 ± 0.32 (1.01–2.05)	4.78 × 10–8***
Intra arterial PI	0.77 ± 0.22 (0.30–1.04)	1.16 ± 0.17 (0.86–1.55)	2.50 × 10–7***
*p*-*values*	*0.06*^*ns*^	0.003**	
Intra Venous PI	0.25 ± 0.16 (0.03–0.70)	0.52 ± 0.16 (0.34–0.97)	3.54 × 10–6***
Extra Venous PI	0.47 ± 0.25 (0.14–0.91)	0.86 ± 0.40 (0.34–1.89)	0.002***
*p-values*	0.001 **	0.0002***	
Heart rate (beats/min)	69 ± 14 (47–95)	70 ± 11 (60–100)	0.72^ns^

In brackets are the maximal (max) − minimal (min) values. Values in italics cells are the *p*-values for the comparisons between extracranial and intracranial levels for each group. Those in underlined are the *p*-values for the comparison between HYV and HEV. *p < 0.05; **p < 0.01; ***p < 0.001

*ns* non-significant, *ACBF* arterial cerebral blood flow, *VCBF* venous cerebral blood flow, *SV* stroke volume, *PI* pulsatility index

### Comparison between flow parameters in HYV and HEV

Results showed that mean ExtraACBF, IntraVCBF, and αExtra were significantly higher in HYV than HEV, whereas Intra blood SV, and all pulsatility indexes were significantly higher in HEV (Table 1). Spinal CSF SV was also significantly higher in HEV (0.51 ± 0.20 ml/cc) than in HYV (0.34 ± 0.21 ml/cc). All the remaining parameters, i.e. IntraACBF, ExtraVCBF, αIntra (HYV: 1.52 ± 0.34 and HEV: 1.59 ± 0.41), Extra blood SV, aqueductal CSF SV, and heart rate were similar in both groups.

In both groups, aqueductal CSF SV represented approximately 10% of spinal CSF stroke volume (respectively 11.76% and 9.80% in HYV and HEV) (Table 1).

#### Correlation between spinal and aqueductal CSF stroke volumes and between CSF stroke volumes and blood stroke volumes

**Fig. 4:**
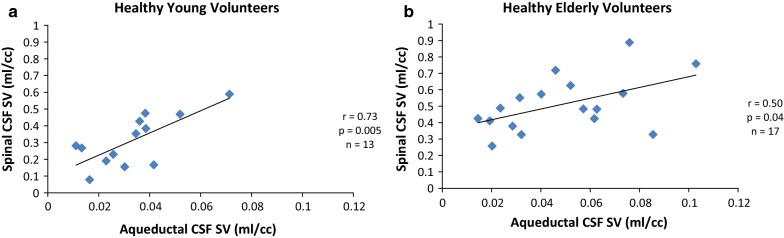
Relationship between cervical and ventricular CSF. Correlation between pulsating cerebrospinal fluid (CSF) stroke volume during one cardiac cycle (in milliliter per cardiac cycle [ml/cc]). The cervical (spinal) CSF stroke volume (SV) is positively correlated to the aqueductal CSF SV and significant, in both healthy young volunteers (**a**) and healthy elderly volunteers (**b**)

There was a significant positive correlation between aqueductal CSF SV and spinal CSF SV in the HYV (r = 0.73; *p* = 0.005). We also found a significant positive correlation between aqueductal CSF and spinal CSF stroke volumes (r = 0.50; *p *= 0.04) in the HEV (Fig. 4).

**Fig. 5:**
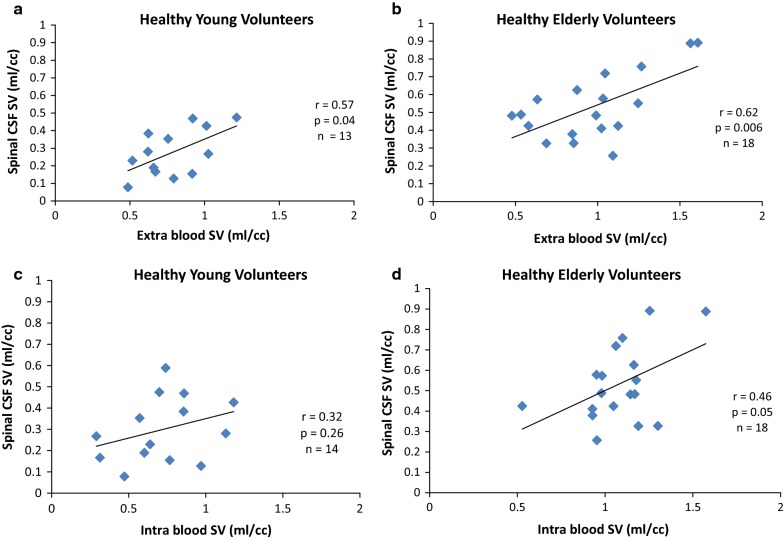
Relationship between cervical (spinal) CSF stroke volume and blood stroke volume. Correlation between oscillating cerebrospinal fluid (CSF) volume during one cardiac cycle (in milliliter per cardiac cycle [ml/cc]) at the cervical level (spinal CSF stroke volume [SV]) and blood stroke volume (SV blood) at intracranial and extracranial levels in the young and elderly. There is a moderate and significant correlation between spinal CSF SV and Extra blood SV in young volunteers (**a**) and in the elderly population (**b**). Spinal CSF SV and Intra blood SV did not correlate in young subjects (**c**) or in elderly subjects (**d**)

For the relation between CSF stroke volumes and blood stroke volumes, we found a significant positive correlation between spinal CSF SV and Extra blood SV in both groups (HYV: r = 0.57; *p *= 0.04 and HEV: r = 0.62; *p *= 0.006). However, at intracranial level and in both groups, spinal CSF SV did not correlate with Intra blood SV (HYV: r = 0.32; *p* = 0.26 and HEV: r = 0.46; *p* = 0.05) (Fig. 5).

## Discussion

Our study showed that aging induced a significant decrease in ACBF and IntraVCBF with little involvement of peripheral veins in extracranial venous drainage. Our elderly population exhibited a significant increase in spinal CSF stroke volume, whereas aqueductal CSF stroke volume remained unchanged with age. Arterial and venous blood pulsatilities were higher in the elderly population than in the young population. Both populations showed higher blood flow pulsations in jugular veins than in the sinuses. When comparing the extracranial and intracranial levels in young subjects, we found that, only the pulsatility of the venous flow changed and not the arterial pulsatility. For the HEV, both arterial and venous pulsatility decreased at the intracranial level. In young and elderly subjects, CSF flow and vascular blood flow were positively correlated at extracranial level but not at intracranial level. As many idiopathic diseases, such as hydrocephalus, intracranial hypertension, and Chiari malformation are related to hydro-hemodynamic alterations, PC-MRI investigations should help to highlight potential flow and pulsatility disturbances in such patients.

### Changes in intra- and extracranial arterial cerebral blood flow patterns

In this study, arterial PI and venous PI provided a general view of cerebral hemodynamics. In the young population, we found that the extracranial arterial PI (0.85 ± 0.17) was similar to the previous value (0.84 ± 0.13) reported by Zarrinkoob et al. [8] and that intracranial arterial PI (0.77 ± 0.22) was also comparable to that mentioned in a previous work (0.81 ± 0.21) [24]. In the elderly population, the extracranial arterial PI value (1.31 ± 0.32) was higher than that previously reported (0.96 ± 0.15) [8]. Inside the cranium, the decreased blood flow pulsatility in both populations is consistent with the literature [6, 8] and could be related to the Windkessel effect, to the geometry, to the compliance of vessels or to the small amount of compliance of brain tissue.

The existence of brain tissue compliance is questioned. It is thought that the brain might not have any compliance because of its high-water content and that water is incompressible. However, we think that intracranial compliance is composed of five main components: arterial compliance, venous compliance, compliance of CSF compartments, intracranial volume compliance and brain tissue compliance. It is not currently possible to estimate directly in vivo the compliance of brain tissue. Nevertheless, we hypothesize that despite its high content of water, the brain tissue as the walls of CSF and vessel compartments would have at least a small compliance. Moreover, during the short period of the cardiac cycle, the Monro-Kellie doctrine is not fully validated since cerebral blood volume expansion is not completely compensated by the intracranial CSF flow toward the spinal canal. Hence, different possibilities may arise: first, the brain tissue is compressible; second, the intracranial volume increases; third, the intracranial volume moves into the spinal canal. This first hypothesis is supported by Voyiadjis and Samadi-Dooki’s work, where the authors demonstrated that only an Ogden hyperelastic model can predict the mechanical behavior of brain tissue in tension and compression [25]. However, in this study we are not able to conclude if there was an increase of intracranial volume or a compression of the brain tissue or a mix of both situations. It is worthwhile to note that the vascular expansion (~ 0.5 ml) is extremely minimal for 1000 ml of the brain volume.

### Changes in intra- and extracranial venous cerebral blood flow patterns

Based on the results of Stoquart-ElSankari et al. extracranial alpha venous correction was 1.66 in healthy young volunteers and 1.65 in healthy elderly volunteers [5]. Qvarlander et al. reported an extracranial alpha venous correction of 1.34 in healthy elderly population [26]. The alpha venous correction factors reported here were comparable in the young population to findings [5] and lower than the results of Qvarlander et al. [26]. However, the values were calculated with the same methodology used by many research teams [26, 27] and the subjects were also comparable based on the age. Based on our results, the percentage of arterial inflow that flows through the sinuses was basically the same in young and elderly subjects (respectively 71% and 64%). In contrary, at extracranial level, there is a great difference between young and elderly subjects for the arterial inflow that flows through the jugular veins (63% vs 90% respectively in HYV and HEV). We don’t have clear and robust explanation to these results. However, different hypotheses could be proposed. Previously, Fall et al. showed that, in young healthy subjects, there is a negative correlation between the total venous flow and the overall sinus resistance of the venous sinus tree [28]. Brown and Thore reviewed the cerebral microvascular pathology in aging and neurodegeneration and found that collagen deposition in veins inhibits blood flow [29]. Further, reduction of sinus area with age has been found in hydrocephalus patients [30]. Here, our study seems to reflect that there is no relative change in sinus resistance during aging. This study also indicates that the contribution of peripheral veins at intracranial level is the same independent of age. Nevertheless, the high participation of jugular to extracranial venous drainage in elderly subjects (90%) seems to reflect an alteration of cervical peripheral venous system pathway or less contribution of extracranial peripheral drainage pathways in lying position in elderly adults. The fact that the sinus flow was 29% lower than jugular flow in the elderly population seems to indicate that apart the sinuses and the peripheral veins drainage system, the contribution of the extra-cerebral veins is not so negligible. Another possible explanation is the existence of a complex cranio-spinal vein system rich in anastomoses, extending from the intracranial and intravertebral veins to the neck and the chest described previously [31]. This was also demonstrated in the last decades and the postural-related changes in cerebral venous system have been highlighted [10, 32]. Our study emphasizes the fact that there is a real difference in venous outflow, and highlights the important role of the peripheral venous pathways.

Pulsatility of venous flow decreases inside the cranium irrespective of the age, as reported previously [10]. Physiologically, sinuses are more rigid than jugular veins because the sinuses are located between two rigid layers of dura mater. This prevents the compression of sinuses when intracranial pressure (ICP) rises. In contrast, jugular veins are largely compliant due to the superficial tissues of the face and neck [33]. Bateman suggested that the arterial brain expansion is dissipated by a combination of CSF movement out the skull and venous compression [34]. The cortical venous compression would therefore be related to the rise of intracranial pressure due to the arterial inflow. Following this assumption, pulsations are transmitted from the arterial system to capillaries, and thereafter to the venous system, mainly governed by the ICP. If this theory holds, we would expect that the pulsatility in sinuses would be greater than the pulsatility in jugular veins. However, we found higher venous pulsatility in jugular veins than in sinuses in both groups (Table 1). In our opinion, the main origin of blood pulsatility in cerebral veins and sinuses is the cardiac aspiration when the tricuspid valve is opened, as also pointed out previously [35, 36].

High flow organs such as the brain and kidneys are particularly sensitive to excessive pressure and flow pulsatility [37]. Levy Nogueira et al. reported that brain mechanical stress refers to external pressure, hydro-hemodynamics stress and viscoelastic stress exerted against the brain [38]. In the rigid the cranium, arterial pressure is the main contributing intracranial factor for brain mechanical fatigue during lifespan. Excessive pressure and flow pulsatility are associated with microvascular remodelling that increases resting resistance and limits hyperaemic reserve [39]. The concordant increase in flow pulsatility and cerebrovascular resistance limits mean flow and results in additive effects on pulsatility index. In the two populations, the decrease of arterial and venous pulsatility inside the cranium could thus be related to the protection of the brain.

### Effect of age on blood and CSF flow patterns

Aging is accompanied by a decrease in gray and white matter [15], so there is less structure to supply oxygen. The reduction in arterial cerebral blood flow in the aging population is in accordance with this fact, and with the previous reported values [5]. With aging, vessels become stiffer [8]. Because tortuosity of vessels has no effect on mean flow [40] and a small effect on pulsatility, the increase in blood pulsatility, as observed during aging, could be related to the decrease of vascular tree compliance.

During cardiac cycle, intracranial brain pulsatility results from: (a) an increase of intracranial volume that leads to centrifugal forces in direction of the subarachnoid spaces; (b) an increase of intracranial volume that leads to centripetal forces in direction of the ventricles; (c) a potential very small compression of the brain volume; (d) a potential small to and fro displacement of brain tissue in direction of the spinal canal in normal conditions and a rise of brain tissue pulsatility in pathological conditions such as Chiari malformation. These events occur to prevent a pathological rise of ICP. CSF flushing from ventricular system and subarachnoid spaces depends on the anatomy of the subject and the resistance to flow. In both studied samples, and as reported previously [1], aqueductal CSF stroke volume represents only 10% of spinal CSF stroke volume. Moreover, the values of aqueductal and spinal CSF SV and also blood SV are in range of previously reported values [1, 7]. Vascular stroke volume is related to cervical CSF oscillations. Assuming that (i) resistance to flow is constant in healthy volunteers’ ventricular and subarachnoid compartments and (ii) pressure difference does not vary between both compartments, it is plausible that spinal CSF variability is related to ventricular CSF variability. However, in diseases such as hydrocephalus for which ICP amplitude increases [41, 42], we would expect that the relation between ventricular and spinal CSF pulsatility would change. Given the variation of cerebral blood volume expansion (blood SV) among subjects, we propose that CSF flow be studied both in spinal and ventricular compartments, but it is also crucial to take into account cerebral blood volume expansion to better explain cranio-spinal dynamics.

## Limitations

Our study is limited by the small sample size of healthy young volunteers and healthy elderly volunteers. These small samples could have impacted our results and the presence or absence of hypertension could be an issue. Some authors showed recently that the antihypertensive combination therapy was prescribed more often to older than to young adults [43]. As certain elderly volunteers were treated with antihypertensive medication and because the young adults that did not report hypertension antecedent or receive any medication, we estimated that both groups were free from arterial hypertension. We also think that the pulsatility index is not affected by our approach because both samples did not present blood flow alteration. Further, by calculating one pulsatility index for the cerebral arterial tree and another for the cerebral venous tree, rather than assessing a single PI for each vessel, we considered that PI is homogenous in the different vessels without any shift of maximal and minimal flows in the different vessels. This also represents a limitation in this study. It has been shown that CSF flow dynamics parameters measured in the cerebral aqueduct are partly age and sex dependent [44]. Another study showed that that body size influences blood pressure and velocity regulation in the young population [45]. Here, we did not take into account the effects of blood pressure, gender or height on the vascular-CSF flows dynamic. We nevertheless think that these parameters would not greatly influence our results.

## Conclusion

We demonstrated that blood flow pulsatility is lower in intracranial compartment compared to the extracranial compartment irrespective of age. Interactions between vascular and CSF are preserved during aging. Our study showed that the multiple pulsatility-related parameters of CSF and cerebral blood flow differ between older and younger adults.

## Data Availability

The part drawings of the constructional design are available from the corresponding author on request.

## Abbreviations

αExtra: extracranial alpha venous corrector factor
αIntra: intracranial alpha venous corrector factor
Aqueductal CSF SV: aqueductal CSF stroke volume
CSF: cerebrospinal fluid
ExtraACBF: extracranial arterial cerebral blood flow
ExtraVCBF: extracranial venous cerebral blood flow
Extra Arterial PI: extracranial arterial pulsatility index
Extra blood SV: extracranial blood stroke volume
Extra Venous PI: extracranial venous pulsatility index
IntraACBF: intracranial arterial cerebral blood flow
Intra blood SV: intracranial blood stroke volume
HEV: healthy elderly volunteers
HYV: healthy young volunteers
ICP: intracranial pressure
PI: pulsatility index
SV: stroke volume
Spinal CSF SV: spinal CSF stroke volume
Intra Arterial PI: intracranial arterial pulsatility index
Intra Venous PI: intracranial venous pulsatility index
ml/cc: milliliter/cardiac cycle
ml/min: milliliter/minute
